# Parental Decision-Related Factors Are Associated with Discretionary Ultra-Processed Food Consumption Among Children and Adolescents Living in the Mediterranean Area

**DOI:** 10.3390/nu18132128

**Published:** 2026-07-01

**Authors:** Francesca Giampieri, Alice Leonardi, Giuseppe Di Costanzo, Tania Abril-Mera, Alice Rosi, Evelyn Frias-Toral, Achraf Ammar, Raynier Zambrano-Villacres, Osama Abdelkarim, Mohamed Aly, Juancho Pons, Laura Vázquez-Araújo, Fernando Maniega Legarda, Alessandro Scuderi, Nunzia Decembrino, Ana Mata, Adrián Chacón, Pablo Busó, Fabio Galvano, Marialaura Bonaccio, Giuseppe Grosso

**Affiliations:** 1 Dipartimento di Scienze Cliniche Specialistiche e Odontostomatologiche, Università Politecnica delle Marche, Via Ranieri, 60131 Ancona, Italy; 2 Joint Laboratory on Food Science, Nutrition, and Intelligent Processing of Foods, Polytechnic University of Marche, Italy, Universidad Europea del Atlántico Spain and Jiangsu University, China at the Polytechnic University of Marche, 60131 Ancona, Italy; 3 Research Group on Food, Nutritional Biochemistry and Health, Universidad Europea del Atlántico, Isabel Torres 21, 39011 Santander, Spain; 4 Department of Biomedical and Biotechnological Sciences, University of Catania, 95123 Catania, Italy; 5 Research Unit of Epidemiology and Prevention, IRCCS Neurological Institute of Southern Italy NEUROMED, 86077 Pozzilli, Italy; 6 School of Medicine, Universidad Católica de Santiago de Guayaquil, Guayaquil 090615, Ecuador; 7 Human Nutrition Unit, Department of Food and Drug, University of Parma, 39 Via Volturno, 43125 Parma, Italy; 8 Escuela de Medicina, Universidad Espíritu Santo, Samborondón 0901952, Ecuador; 9 Department of Training and Movement Science, Institute of Sport Science, Johannes Gutenberg-University Mainz, 55122 Mainz, Germany; 10 High Institute of Sport and Physical Education of Sfax, University of Sfax, Sfax 3000, Tunisia; 11 Research Laboratory, Molecular Bases of Human Pathology, LR19ES13, Faculty of Medicine of Sfax, University of Sfax, Sfax 3000, Tunisia; 12 Department of Nutrition and Food Technology, School of Agriculture, The University of Jordan, Amman 11942, Jordan; 13 Facultad de Ciencias de la Salud y Desarrollo Humano, Universidad Ecotec, Km. 13.5 Samborondón, Samborondón, EC092302, Ecuador; 14 Faculty of Sport Sciences, Assiut University, Assiut 71515, Egypt; 15 Department of Sports Management, School of Business, ESLSCA University, Giza 12676, Egypt; 16 Institute of Sports and Sports Science, Karlsruhe Institute of Technology, 76131 Karlsruhe, Germany; 17 Faculty of Liberal Arts and Sciences, Chukyo University, Nagoya 466-0825, Japan; 18 Editorial Luis Vives (EDELVIVES), Carretera de Madrid, 50012 Zaragoza, Spain; 19 BCC Innovation, Technology Center in Gastronomy, Basque Culinary Center, 20009 Donostia-San Sebastián, Spain; 20 Basque Culinary Center, Faculty of Gastronomic Sciences, Mondragon Unibertsitatea, 20009 Donostia-San Sebastián, Spain; 21 Faculty of Health Science, Universidade Internacional do Cuanza, Cuito EN250, Angola; 22 Faculty of Health Science, Universidad de La Romana, La Romana 22000, Dominican Republic; 23 Department of Agriculture, Food and Environment, University of Catania, Via S. Sofia 100, 95123 Catania, Italy; 24 Neonatal Intensive Care Unit, Integrated Department for Maternal and Child’s Health Protection, University Hospital “Policlinico-San Marco” Catania, 95100 Catania, Italy; 25 Technological Institute for Children’s Products & Leisure AIJU, 03440 Alicante, Spain

**Keywords:** ultra-processed foods, barriers and enablers, parental attitudes, food literacy, dietary behaviors, nutritional transition, eating habits, children, adolescents

## Abstract

**Background/Objectives**: Nutrition during childhood and adolescence is a key determinant of long-term health, influencing metabolic homeostasis, neurocognitive development, and immune system maturation. Globalization and technological advances have reshaped food production and consumption, increasing the availability of ultra-processed foods (UPF) of low nutritional quality. This study aimed to investigate the relationship between parental factors, namely food literacy, perceived barriers and enablers, dietary attitudes, and healthy eating behaviors, and the consumption of discretionary UPF among children and adolescents living in 5 Mediterranean countries. **Methods**: This cross-sectional study was based on a survey completed by 2011 parents of children and adolescents aged 6–17 years from 5 Mediterranean countries, who reported on their children’s dietary and lifestyle habits. Adherence to the Mediterranean diet was assessed through the KIDMED index. Parental food literacy was measured using the Short Food Literacy Questionnaire (SFLQ). Perceived barriers and enablers were assessed based on the Theory of Planned Behavior, and parents’ attitudes toward their child’s diet were evaluated using the Healthy-Eating Attitudes Questionnaire (HEAQ). Finally, the Theory of Internet Use Related to Health (TIUH) questionnaire was used to assess parents’ tendencies related to health information use online. **Results**: Higher perceived barriers and enablers were significantly associated with lower discretionary UPF consumption across all models. Parental food literacy (SFLQ) showed a positive association with discretionary UPF consumption, remaining significant in the fully adjusted model, although with reduced magnitude. Healthy-eating attitudes (HEAQ) were initially positively associated with discretionary UPF intake but lost statistical significance after full adjustment. Regarding health-related internet use (TIUH), the Health Information dimension showed a strong positive association with discretionary UPF consumption, while other dimensions (Consciousness and Beliefs) showed inconsistent and non-significant associations in the fully adjusted model. **Conclusions**: Children’s consumption of discretionary UPF is shaped by several interrelated factors, such as family environment, eating patterns, and parents’ perceptions, rather than solely by knowledge or attitudes.

## 1. Introduction

Nutrition during the developmental age represents an important determinant of global public health, exerting long-lasting effects on metabolic homeostasis, neurocognitive development, and immune system maturation [1,2,3]. Childhood and adolescence constitute periods of particular biological and behavioral vulnerability, during which food preferences and dietary habits are shaped by the dynamic interaction between genetic factors and environmental, social, and cultural influences [4,5]. This process is profoundly affected by the so-called nutritional transition, characterized by the progressive abandonment of traditional dietary models, including the Mediterranean diet, in favor of Westernized eating patterns marked by high energy density and low nutritional value, not only among younger individuals but also among adults [6,7]. This shift, closely linked to globalization and technological advancement, has been profoundly influenced by food production practices, promoting the widespread availability of industrial products of low nutritional quality [8]. Higher nutrient-poor, energy-rich food consumption has been deemed largely responsible for the global trends in childhood obesity and the rise in adult non-communicable disease rates [9].

According to the Nova classification proposed by Monteiro and colleagues, ultra-processed foods (UPF) are industrial formulations predominantly derived from food fractions and additives, with minimal or no presence of whole foods [10]. The organoleptic properties of UPF, enhanced through advanced industrial processing techniques, confer high hyper-palatability, thereby promoting excessive and habitual consumption at the population level [11]. These features are associated with significantly reduced costs, paired with the sensory appeal, amplified by cosmetic modifications and flavorings, that ultimately facilitates their widespread diffusion among young populations [11]. A large share of discretionary UPFs (i.e., sugary drinks, confectionery, chips, fast food, pastries) is inherently “unhealthy” as they are characterized by a suboptimal nutritional quality as compared to other ultra-processed formulations that may still retain adequate nutritional content (such as yogurts, whole-grain cereals, etc.) [12]. From a biochemical perspective, most UPFs exhibit a profoundly imbalanced nutritional profile, being rich in sodium, added sugars, and excess saturated fats, while being severely depleted in essential nutrients such as fiber, proteins, vitamins, and minerals [13]. This imbalance extends beyond the individual food item and affects overall dietary patterns: individuals with high discretionary UPF consumption tend to adopt less healthy eating behaviors, showing higher total energy intake, lower fiber consumption, and an overall poorer nutrient profile, characterized by imbalances in both macro- and micronutrient intake, along with reduced adherence to healthy dietary patterns [14,15]. A growing body of scientific evidence links high UPF intake during childhood to being overweight, having obesity and cardiometabolic comorbidities [16], and alterations in psychological well-being, such as food addiction, depressive symptoms and anxiety [17,18]. Current scientific debate seeks to clarify whether these adverse health outcomes are attributable solely to poor nutritional quality or whether industrial processing techniques and the use of additives exert an independent and yet incompletely understood role [19].

The family has been suggested to play a pivotal role in mediating children’s exposure to an obesogenic environment [20,21]. The literature shows that children’s dietary choices are not determined solely by economic availability, but also by behavioral models through which they internalize habits and lifestyles that persist over time [22]. Parental capacity to promote healthy dietary choices is, however, a complex and multidimensional process, strongly influenced by factors such as parental food literacy, defined as the set of knowledge, skills, and practical competencies required to critically interpret the food system and counteract industrial marketing pressures [23]. Nevertheless, the translation of nutritional knowledge into concrete behaviors is not automatic and is often mediated by perceived barriers and facilitators [24]. Factors such as limited time availability, work-related stress, and economic constraints may hinder the adoption of healthy eating practices, even in the presence of adequate awareness [25]. Moreover, dietary determinants evolve substantially across childhood and adolescence [26]. During early childhood, parents exert considerable influence over food consumption through food availability, feeding practices, role modelling, and control of the home food environment [27,28,29]. However, as children mature, increasing autonomy and independent decision-making reduce direct parental control over eating behaviours [30]. Adolescents are progressively exposed to a wider range of influences, including peer norms, school and out-of-home food environments, food marketing, social media content, and opportunities to independently purchase foods [31]. Consequently, dietary choices during adolescence reflect a more complex interplay between family, social, environmental, and individual factors [32]. Nevertheless, parental influences may remain relevant through the establishment of long-term eating habits, household food availability, and broader family norms surrounding food consumption [33]. It therefore becomes relevant to explore the coherence between the cognitive dimension, represented by attitudes toward healthy eating, and the behavioral dimension, expressed through everyday dietary practices [34]. In light of these considerations, the present study aims to examine the association between parental psychosocial factors and the consumption of discretionary UPF among children and adolescents, through the analysis of parental food literacy, perceived barriers, as well as dietary attitudes and behaviors.

## 2. Materials and Methods

### 2.1. Study Population

This study is a cross-sectional analysis conducted within the framework of the DELICIOUS project and involved a survey of parents reporting on the dietary and lifestyle habits of children and adolescents aged 6–17 years living in five Mediterranean countries (Italy, Spain, Portugal, Egypt, and Lebanon) [35]. Participants were recruited on a voluntary basis using a consumer database established by one of the study partners and were required to be parents of children within the target age range and to have internet access. Based on recent literature with similar objectives and methodologies, a target sample size of approximately 400 participants per country was considered sufficient to detect meaningful differences in adherence to the Mediterranean diet and its associated determinants [36,37,38,39,40]. Data were collected using an electronic survey, resulting in a final sample of 2011 participants. The primary aim of this preliminary survey was to assess adherence to the Mediterranean diet and associated factors among children and adolescents in the participating countries. All study procedures were conducted in accordance with the World Medical Association’s Declaration of Helsinki (1989), and informed consent was obtained from all participants prior to study participation.

### 2.2. Background Information

Information on participants’ demographic characteristics and lifestyle factors was collected through the survey. For parents, data regarding sex, age, educational attainment, and occupational status were recorded, whereas for children and adolescents, sex, age, and anthropometric parameters were collected. Children’s age was grouped into two categories (6–11 years and 12–17 years). Parental educational level was classified into three categories: low (primary education), medium (secondary education), and high (tertiary education). Body mass index (BMI) for children and adolescents was calculated using self-reported weight and height and categorized according to the percentile cut-offs of the Centers for Disease Control and Prevention (CDC) growth charts for individuals aged 2–19 years [41]. Based on these criteria, participants were classified as normal weight (BMI 5th–84th percentile), overweight (BMI 85th–94th percentile), or obese (BMI ≥ 95th percentile). Physical activity was assessed using the International Physical Activity Questionnaire–Short Form (IPAQ) [42], which evaluates walking, moderate-intensity, and vigorous-intensity activities performed over the previous seven days, including frequency and duration. Physical activity levels were subsequently categorized as low, moderate, or high in accordance with IPAQ scoring guidelines. Sleep duration was classified following the National Sleep Foundation recommendations [43] into three categories: less than 8 h, 8–10 h, and more than 10 h per day. Daily screen time was categorized as less than 2 h, 2–4 h, or more than 4 h per day. Eating habits including breakfast habits, with whom and where the main meals are consumed, and home-cooking frequency were recorded via specific questions.

### 2.3. Mediterranean Diet Adherence

To assess overall diet quality, the Mediterranean Diet Quality Index (KIDMED) was applied to the collected dietary data [44]. The KIDMED score consists of a structured questionnaire comprising 16 questions regarding food items in line (or misaligned) with the Mediterranean dietary pattern, to which positive or negative scores are assigned, for a maximum total score of 12, with a score ≥ 7 deemed as having high adherence to the Mediterranean diet.

### 2.4. Discretionary UPF Consumption

The average weekly food intake was assessed through questions regarding the frequency of consumption of key food groups of interest. The term “discretionary UPF” was used to describe a subset of ultra-processed products characterized by low nutritional quality and traditionally referred to as “junk foods” in the literature. The questions used in the food frequency questionnaire aimed to explore the frequency of consumption of discretionary, non-core foods, which are nowadays almost universally classified as UPFs. The question addressed the food groups as follow: “How many times per week does your son or daughter consume ultra-processed foods (these are those that receive an industrial treatment during which the nature of the food is modified)” listing the following food groups: processed meats (i.e., hot dogs), industrial pizza, industrial cookies, commercial sauces (i.e., ketchup, mustard, barbecue sauce, etc.), pastries (i.e., confectionaries), ready meals (i.e., frozen or canned pasta), processed fish (i.e., frozen nuggets), salted and smoked meats (i.e., some types of reconstructed cured meats), sweets (i.e., industrial ice-creams), soft drinks, fried foods (i.e., fast food chips), grilled foods (i.e., fast food burgers), and other fast foods (i.e., chicken nuggets). Frequency of consumption was then categorized as “high” or “low” using the median intake as the threshold.

### 2.5. Psychosocial Assessment

Parental food literacy was evaluated using the Short Food Literacy Questionnaire (SFLQ) for adults [45], a validated instrument covering multiple aspects of food literacy, including functional and interactive competencies. The questionnaire contains 15 items distributed across nine domains and employs either four- or five-point Likert-type response scales. Depending on the item, response categories range from “very bad” to “very good,” “strongly disagree” to “strongly agree,” “very difficult” to “very easy,” “very hard” to “very easy,” or “never” to “always.” Ten items assess practical food literacy skills, such as interpreting nutrition information and planning nutritionally balanced meals. Interactive food literacy is measured by one item addressing the exchange of nutrition-related information with family members or peers. The remaining four items evaluate critical food literacy, including the appraisal of nutrition information and the assessment of long-term health consequences associated with dietary choices. Total scores range up to 62 points, with higher scores reflecting greater levels of food literacy.

Perceived barriers and enablers (PBE) related to healthy eating were measured using a questionnaire developed according to the Theory of Planned Behavior (TPB) framework [46]. TPB proposes that behavioral intentions are shaped by three principal constructs: attitudes toward the behavior, subjective norms, and perceived behavioral control. Attitudes refer to an individual’s positive or negative evaluation of a behavioral change, subjective norms capture perceived social expectations from significant others, and perceived behavioral control reflects the perceived ease or difficulty of adopting the behavior. Drawing on previous research [47,48], a three-section instrument was designed to assess parents’ perceived facilitators and obstacles to maintaining a healthy diet. The first section includes 14 items evaluating attitudes toward healthy eating. Responses are recorded on a five-point Likert scale ranging from “strongly disagree” (1 point) to “strongly agree” (5 points), with an additional “do not know/no answer” option scored as 0. The second section consists of four items assessing subjective norms, using a five-point scale ranging from “highly unlikely” (1 point) to “highly likely” (5 points), together with the same “do not know/no answer” option. Perceived behavioral control is assessed through a single item in the third section, scored using the same response format as the subjective norms domain. The maximum attainable score for the overall questionnaire is 95 points.

Parents’ attitudes toward the quality of their child’s diet were assessed using the Healthy-Eating Attitudes Questionnaire (HEAQ) [49]. This instrument comprises eight items addressing parental views regarding their child’s consumption of fruit, vegetables, dietary fibre, fish, butter, fat, meat, sweets, and pastries. Responses are dichotomous (“yes” or “no”), with affirmative answers assigned 1 point and negative answers assigned 0 points. Total scores can therefore range from 0 to 8, with higher scores indicating more favorable healthy-eating attitudes.

Health consciousness and online health-information seeking behaviors were evaluated using the Theory of Internet Use Related to Health (TIUH) questionnaire [50]. This multidimensional instrument examines parents’ propensity to use internet resources for health-related purposes and consists of four domains. The health-information domain identifies the types of health topics searched online and uses dichotomous (“yes/no”) response options. The health consciousness domain contains five items rated on a five-point scale ranging from “strongly disagree” to “strongly agree.” Health-information orientation is assessed through eight items scored on the same five-point scale. Finally, the health-oriented beliefs domain includes eight items evaluating perceptions and beliefs related to health behaviors, also using five-point response categories. Maximum scores of 6, 25, 40, and 40 points can be achieved for the health-information, health-consciousness, health-information orientation, and health-oriented beliefs domains, respectively.

### 2.6. Statistical Analysis

Descriptive statistics were used to summarize the study variables. Categorical data are reported as absolute frequencies and percentages, and differences between groups were examined using the chi-square test. Continuous variables are presented as mean values with their corresponding standard deviations (SD). The distributional properties of continuous variables were assessed by evaluating normality and homogeneity of variance using the Kolmogorov–Smirnov and Levene tests, respectively.

For comparisons involving continuous variables, parametric tests were applied when assumptions of normality were satisfied. Specifically, Student’s *t*-test was used for comparisons between two groups, whereas one-way analysis of variance (ANOVA) was employed for comparisons across multiple groups. When the assumptions required for parametric testing were not met, non-parametric alternatives were adopted, namely the Mann–Whitney U test and the Kruskal–Wallis test.

Associations between weekly consumption of discretionary UPFs and psychosocial measures were initially explored through univariate linear regression analyses. Predictor variables included scores derived from the SFLQ, PBE, HEAQ, and TIUH instruments. Subsequently, multivariable linear regression models were fitted to examine these relationships while accounting for potential confounders. Regression coefficients (β) and their standard errors (SEs) were estimated after adjustment for child-related characteristics (age, sex, body mass index, physical activity level, sleep duration, and screen time) and parental characteristics (age, sex, educational attainment, household income, family structure, and residential area).

To evaluate potential collinearity among independent variables, multicollinearity diagnostics were conducted, including variance inflation factors (VIFs), tolerance statistics, and correlation analyses. Statistical significance was established at a two-sided *p*-value < 0.05. All analyses were performed using IBM SPSS Statistics version 29 (IBM Corp., Armonk, NY, USA).

## 3. Results

Based on Table 1, the mean weekly consumption of discretionary UPF differed significantly across demographic characteristics and lifestyle factors among children and adolescents. Adolescents aged 12–17 years reported a higher mean UPF intake compared with children aged 6–11 years. A significant difference was also observed by sex, with females showing a greater mean discretionary UPF consumption than males (*p* < 0.001). UPF intake increased progressively across weight status categories: normal-weight children reported the lowest consumption, whereas higher intakes were observed among overweight and obese participants, with statistically significant differences between groups (*p* < 0.001). Significant associations were also found between discretionary UPF consumption and sleep duration: children sleeping 8–10 h per night had lower mean UPF intake compared with those sleeping less than 8 h or more than 10 h (*p* < 0.001). Similarly, discretionary UPF consumption was strongly associated with screen time, increasing with longer daily exposure. Participants reporting less than 2 h of screen time per day consumed fewer discretionary UPFs compared with those reporting 2–4 h or more than 4 h per day (*p* < 0.001). Finally, differences in UPF intake were observed across physical activity levels (*p* < 0.001). Children with low physical activity reported the lowest mean consumption, while higher intakes were observed among those with medium and high physical activity levels.

As reported in Table 2, children’s weekly intake of discretionary UPF showed significant variation according to several parental demographic characteristics. Higher discretionary UPF consumption was observed among children of younger parents aged 44 years or less compared with those whose parents were 45 years or older, and this difference was statistically significant (*p* < 0.001). No meaningful differences in discretionary UPF intake were found in relation to parental sex. In contrast, parental educational attainment was significantly associated with children’s discretionary UPF consumption (*p* < 0.001). Children of parents with a higher level of education exhibited greater weekly discretionary UPF intake compared with those whose parents had medium or low education levels. Family structure was also significantly related to discretionary UPF consumption: higher intake was observed among children living with a single parent or with others, whereas lower consumption was reported among those living with two parents. Household income showed a clear positive association with discretionary UPF intake, with consumption increasing across income categories and reaching the highest levels among families with higher incomes. Finally, the area of residence was significantly associated with discretionary UPF consumption. Children living in urban settings reported higher weekly discretionary UPF intake compared with those residing in rural areas, and this difference was statistically significant.

Table 3 shows children’s weekly mean (±SD) consumption of discretionary UPF according to eating habits. Significant differences in discretionary UPF intake were observed across all behaviors analyzed (*p* < 0.001). Regular breakfast consumption was associated with lower discretionary UPF intake: children who always ate breakfast reported lower weekly consumption compared with those who did so often or never/seldom. Similarly, daily family meals were linked to reduced discretionary UPF consumption, whereas higher intakes were observed among children eating with their family often or seldom. Children who rarely ate alone showed the lowest discretionary UPF intake, while those eating alone often or daily reported significantly higher consumption. With respect to eating at school, higher discretionary UPF intake was observed among children who ate at school often compared with those who did so never/seldom or almost daily. Finally, frequent consumption of home-cooked food was inversely associated with discretionary UPF intake, with lower consumption observed among children eating home-cooked meals almost daily compared with those consuming them often.

Table 4 presents mean scores (±SD) of parental food literacy, perceived barriers and enablers, healthy-eating attitudes, and healthy-eating behaviour according to frequency of children’s discretionary UPF consumption. PBE scores were significantly lower in families with high UPF consumption compared with low discretionary UPF consumption (*p* < 0.001). In contrast, SFLQ scores did not differ significantly between low and high discretionary UPF consumption groups. No significant differences were found in HEA scores across discretionary UPF consumption categories. Regarding TIUH scores, the group with higher discretionary UPF consumption had higher scores in the Health Information (*p* < 0.001). In addition, the Consciousness and Beliefs subscales were higher in the low-discretionary UPF consumption group.

Table 5 shows the associations between parental food literacy, perceived barriers and enablers, healthy-eating attitudes, and health-related internet use and children’s weekly consumption of discretionary UPFs. In the unadjusted model (Model 1), higher parental food literacy (measured through SFLQ) and health-related internet use (measured through TIUH) were positively associated with children’s UPF consumption, whereas perceived barriers and enablers (measured through PBE) showed a significant inverse association. Healthy-eating attitudes (measured through HEA) were also positively associated with discretionary UPF intake. Among TIUH dimensions, health information consciousness and orientation were positively associated, while beliefs showed an inverse association. After adjustment for children’s sociodemographic and lifestyle characteristics (Model 2), the associations for SFLQ, PBE, HEA, and TIUH scores remained significant, with a stronger positive association observed for HEA scores. In Model 3, further adjustment for children’s eating habits did not substantially change the results; SFLQ, PBE, HEA, and TIUH scores remained significantly associated with discretionary UPF consumption, and the beliefs dimension of TIUH became positively associated. In the fully adjusted model (Model 5), including adherence to the Mediterranean diet, parental food literacy remained positively associated with discretionary UPF consumption, although with reduced magnitude, while PBE scores maintained a robust inverse association. TIUH scores continued to show a strong positive association, whereas HEA scores were no longer statistically significant. VIF and tolerance values for multicollinearity diagnostics indicated no evidence of problematic collinearity among the predictors, with all VIF values below 3 and tolerance values above 0.3. Correlation matrices showed most r values below 0.1 with only some below 0.5 suggesting related but distinct constructs.

## 4. Discussion

The present study provides a comprehensive analysis of parental decision-related factors associated with discretionary UPF consumption among children and adolescents living in the Mediterranean area, highlighting the complex interplay between early-life dietary patterns and parental education and behaviors.

Parental food attitudes demonstrated a significant association with children’s dietary behaviors. Specifically, the perceived barriers and enablers dimension was inversely correlated with the consumption of discretionary UPF, indicating that parents who perceive fewer obstacles and greater resources for healthy eating are more likely to limit their children’s exposure to these foodstuffs. This finding suggests that, beyond nutritional knowledge, parental self-efficacy and the ability to effectively navigate and manage the food environment play a critical role in shaping children’s dietary patterns [51]. A scoping review aiming to identify the main barriers and facilitators associated with public policies designed to reduce UPF consumption highlighted the central role of structural-level determinants, in particular factors related to governmental action and civil society engagement, emerging as the most relevant facilitators [52]. Regulatory measures, such as fiscal policies, marketing restrictions, and front-of-pack nutrition labeling, together with institutional commitments and advocacy initiatives, were identified as key levers for reshaping food environments and promoting healthier dietary patterns at the population level. Conversely, the same review identified food industry strategies as the most pervasive and recurrent barrier across the countries analyzed [52]. As economic stakeholders with a vested interest in the promotion and sale of UPF, food companies frequently adopt practices that may undermine public health efforts, including intensive marketing (especially targeting children), product reformulation strategies that preserve high hyper-palatability, aggressive pricing policies, and lobbying activities aimed at influencing policy-making processes [53,54]. These dynamics generate structural resistance to regulatory change and may delay or weaken the implementation of effective interventions. While strengthening parental food literacy, particularly by reducing perceived barriers and enhancing enabling factors, may contribute to lowering UPF consumption at the household level, sustainable and equitable change requires robust regulatory frameworks capable of counterbalancing industry influence [55]. Addressing the current food environment and reinforcing government-led and civil society-supported initiatives appear essential to achieving meaningful reductions in UPF intake across diverse sociopolitical contexts [56].

Conversely to the perceived barriers and enablers, higher levels of parental food literacy, as measured by the SFLQ, were unexpectedly associated with greater UPF consumption. This finding suggests that knowledge alone does not necessarily translate into healthier choices, particularly in contexts where UPF are convenient, socially accepted, and often perceived as quick, low-effort meal solutions [57]. Additionally, these results indicate that, more than declarative knowledge, the operational factors governing the daily management of family meals, such as time availability, planning capacity, and practical food preparation skills, play a decisive role. When time constraints exist, food choices tend to shift toward options that minimize organizational and decisional effort [58,59,60]. UPFs effectively meet these demands due to their immediate availability, extended shelf life, and minimal preparation requirements, functioning as practical solutions rather than nutritionally intentional choices [61]. From a mechanistic perspective, this reflects the predominance of automatic over reflective decision-making processes: while nutritional literacy operates at a conscious cognitive level, everyday food-related behaviors are largely driven by habits, established routines, and environmental cues [62]. Under conditions of time constraints or stress, these automatic processes tend to favor rapid, habitual decisions, even when such choices contradict established knowledge regarding nutrition and associated health risks. Therefore, merely enhancing parents’ theoretical knowledge without providing tools for practical management, such as strategic meal planning, the development of quick culinary skills, or improving the out-of-home food environment, risks widening the gap between intention and behavior [63,64]. From this perspective, intervention strategies should move beyond simple information-sharing to actively reducing the cognitive effort required to access healthy options, effectively transforming the home environment into a support system that makes the healthy choice the “default choice,” even in the absence of sufficient time or mental resources [65]. In this light, interventions designed to improve parental nutritional literacy must be redefined to encompass these operational competencies; by doing so, they may foster a more resilient and supportive domestic environment, which plays a pivotal role in modulating children’s dietary habits and ensuring long-term nutritional health [66].

The contemporary digital information ecosystem further contributes to the complexity of this scenario. In the present study, health-oriented eating behavior, assessed through the TIUH score, was paradoxically positively associated with UPF consumption, particularly within the “Health Information” and “Orientation” subscales. Although an increasing proportion of consumers appear to be more aware of the adverse health consequences associated with UPF consumption, recent studies emphasize that screen time among the young population relates to higher UPF consumption, suggesting that intense marketing strategies, including digital advertising and celebrity endorsement, continue to influence consumer trust and purchasing intentions [67,68]. In this context, the food industry has capitalized on advances in food technology and increasingly sophisticated communication strategies to develop and promote reformulated UPF, emphasizing the inclusion of components perceived as healthy or the reduction in detrimental nutrients, thereby enhancing their nutritional perception of consumers [69]. At the same time, parents who actively seek nutrition-related information online may be exposed to heterogeneous, and at times contradictory or misleading, content, as well as to promotional strategies portraying UPF as healthy options or downplaying the relevance of nutritional labeling and the overall poor nutritional quality of these products [70]. This phenomenon may be interpreted in light of information overload and reliance on cognitive heuristics: when faced with ambiguous or inconsistent information, parents may depend on simplified cues, such as health claims, packaging features, or brand familiarity, to guide their food choices, inadvertently contributing to the persistence or reinforcement of UPF consumption [71].

Moreover, healthy-eating attitudes were initially positively associated with UPF consumption; however, this relationship disappeared after full adjustment, suggesting that attitudes may influence behavior indirectly through dietary routines, the home food environment, or perceived barriers rather than directly shaping food choices. In mechanistic terms, attitudes represent upstream motivational determinants that require translation into actionable behaviors, such as meal planning, ingredient availability, and modeling healthy eating for children, to impact actual dietary patterns [72]. In the absence of supportive environmental and practical conditions, these attitudes remain latent, insufficient to counteract habitual or convenience-driven food choices, particularly in obesogenic contexts where UPFs are readily accessible, highly palatable, and socially normalized [73]. These findings underscore that the relationship between food literacy, knowledge, attitudes, and dietary behavior is highly mediated by contextual, cognitive, and behavioral mechanisms. Public health interventions aiming to reduce UPF consumption should therefore move beyond knowledge dissemination and favorable attitudes, addressing practical barriers, supporting the application of nutritional knowledge in daily routines, and enhancing critical appraisal of nutrition-related information to facilitate healthier, sustainable family food choices [74].

This study also provided an overview of background characteristics that may be potential confounding factors as determinants of UPF consumption. Adolescents reported higher UPF consumption than younger children, likely due to greater autonomy, increased exposure to advertising, and more frequent eating outside the home [75,76]. According to socioecological and developmental models of health behaviour, the relative importance of determinants of dietary intake changes across developmental stages [77]. While parents represent the primary influence on children’s eating patterns in early life [78], adolescence is characterized by increasing autonomy and expanding social networks, resulting in greater influence from peers, educational settings, commercial food environments, and digital media [79]. Therefore, the association between parental characteristics and UPF consumption may vary according to age, with parental influences potentially attenuated as adolescents gain independence in food-related decisions. Similarly to other studies, UPF intake was positively associated with weight status, supporting evidence linking these foods (characterized by high energy density and low satiety) to increased adiposity and cardiometabolic risk [80,81]. Lifestyle behaviours such as shorter sleep duration, higher screen time, and physical activity levels were also associated with greater UPF consumption, reflecting interconnected behavioural patterns influencing diet [82,83,84]. Family socio-demographic factors further contributed, with higher intake observed among children of younger parents, higher socioeconomic status, and urban or non-traditional households, possibly due to time constraints and greater access to processed foods [85,86]. Finally, healthier dietary routines (such as regular breakfast, family meals, and home-prepared foods) were linked to lower UPF consumption, whereas eating alone or outside the home was associated with higher intake [87]. Previous investigations provided evidence that parental food literacy, perceived barriers, dietary attitudes and behaviors may also influence such eating and lifestyle factors [88]. However, the present analysis included several adjustment models addressing the potential effects of confounding factors. The attenuation of the associations after adjustment for background characteristics suggests that the relationship between parental attitudes, awareness, and beliefs and children’s UPF consumption is likely mediated or confounded by broader socio-demographic and environmental factors. Indeed, while parental attitudes and nutrition knowledge have been shown to positively influence children’s diet quality, their effects are closely intertwined with structural determinants, such as socio-economic status, household environment, and parental dietary behaviours, which are themselves strong predictors of children’s eating patterns [89,90,91]. Furthermore, evidence indicates that parental knowledge and attitudes do not always translate into actual feeding practices, highlighting a gap between cognition and behaviour in real-world settings [92]. This is consistent with studies showing that broader contextual factors (including time constraints, food availability, and family routines) substantially shape children’s dietary intake, often outweighing individual-level determinants [93]. Collectively, these findings support the interpretation that, once these underlying factors are accounted for, parental attitudes alone may have a limited independent effect on children’s UPF consumption, underscoring the importance of considering the wider socio-environmental context in the interpretation of these associations.

To the best of our knowledge, this study is among the few to comprehensively examine the association between children’s and adolescents’ consumption of UPF and a wide range of individual, familial, and parental determinants, including eating habits, parental food literacy, perceived barriers and enablers, and health-related internet use, using a standardized methodological approach. Nevertheless, the findings should be interpreted in light of certain limitations. First, the cross-sectional design of the study does not allow for the establishment of causal relationships, nor does it exclude the possibility of reverse causation. Second, information on children’s dietary intake and eating behaviors, as well as anthropometric measures, was reported by parents, which may have introduced reporting bias. In addition, the use of food frequency-based measures is inherently subject to recall bias and social desirability bias and may lead to under- or overestimation of actual dietary intake. However, these tools remain widely used and represent the most feasible and accepted instruments in nutritional epidemiology, given the lack of alternative methods with comparable applicability in large population-based studies. Also, pooling participants from five countries may mask country-specific cultural, environmental, policy-related, or dietary influences, and the residual country-level heterogeneity cannot be excluded. Third, the sample selection was convenient and may suffer from selection bias by overrepresenting higher socio-economic status and digitally connected families, thereby limiting generalizability. However, such bias is often observed in this type of study, while on the other hand, the inclusion of participants with a higher cultural level could provide more reliable and valid information. Another limitation of the present study concerns the terminology used to describe children’s consumption of “discretionary” UPFs. The questions used to assess the frequency of consumption of the selected junk foods specifically addressed the level of processing, assuming that industrial formulation and products can be generally considered ultra-processed. Compared to the existing scientific literature on UPFs, most studies do not use FFQs specifically designed to address the level of food processing, while they generally assume the nature of the product based on the type. In the case of the present study, the food products included as exposure are by nature industrial and most unequivocally ultra-processed by formulation design. Although this label was selected because it appropriately captures the nutritional characteristics and health implications of the food products examined in this study, the terminology is not universally standardized or consistently applied across the literature. Other studies may use alternative classifications or definitions for nutritionally inadequate foods undergoing industrial formulations and alterations, which include prepackaged junk foods (i.e., sweets and salty snacks) as well as fast foods (chips and preserved burgers). The findings presented in this study refer to these specific categories of foods and are not to be generalized to the whole landscape of UPFs. Finally, the assessment of UPF consumption was limited to selected discretionary food categories, primarily unhealthy junk foods, and may not fully capture the entire spectrum of UPF consumed as part of the overall diet. The dietary information was not sufficiently detailed to allow accurate estimation of total daily energy intake or the precise energy contribution of UPFs. Nevertheless, the analytical approach adopted in this study was semi-quantitative in nature, as participants were categorized according to relative exposure and frequency of consumption associated with psychosocial factors of parents rather than absolute intake quantities or energy share from UPF as a means of biological effects.

## 5. Conclusions

In conclusion, these findings indicate that discretionary UPF consumption among children and adolescents is driven by multiple interconnected factors, including the family environment, dietary routines, and parental perceptions, rather than by knowledge or attitudes alone. Interventions aimed at reducing discretionary UPF consumption should therefore go beyond nutrition education and address structural and environmental factors, such as promoting regular family meals, increasing the consumption of home-prepared foods, and supporting parents in critically interpreting nutrition-related information. Reducing discretionary UPF consumption among children and adolescents requires a multilevel approach integrating educational strategies, family-based interventions, and environmental modifications. Policies that promote structured eating routines, informed decision-making, greater access to home-cooked meals, and critical media literacy among parents may help mitigate the influence of discretionary UPF.

## Figures and Tables

**Table 1:** Children’s weekly mean (and standard deviation) discretionary UPF consumption by demographic characteristics and lifestyle habits of children and adolescents (*n* = 2011).

	*n* (%)	UPF Consumption (serv/wk)	*p*-Value
		*mean (SD)*	
Age			0.027
6–11 y	1047 (52)	15.8 (9.9)	
12–17 y	964 (48)	16.8 (10.0)	
Sex			<0.001
Male	995 (49)	15.4 (9.3)	
Female	1016 (51)	17.1 (10.5)	
BMI			<0.001
Normal weight	1087 (69)	14.1 (8.2)	
Overweight	263 (17)	18.1 (11.1)	
Obese	232 (15)	20.3 (11.9)	
Sleep duration			<0.001
<8 h	371 (18)	17.5 (10.5)	
8–10 h	1542 (77)	15.7 (9.4)	
>10 h	98 (5)	19.9 (14.6)	
Screen time			<0.001
<2 h/day	1131 (56)	14.1 (8.6)	
2–4 h/day	723 (36)	18.9 (10.7)	
>4 h/day	157 (8)	19.9 (11.5)	
Physical activity level			<0.001
Low	1017 (51)	15.4 (9.1)	
Medium	461 (23)	17.8 (10.7)	
High	533 (27)	16.7 (10.6)	

Abbreviations: Body Mass Index (BMI); Ultra-Processed Foods (UPFs).

**Table 2:** Children’s weekly mean (and standard deviation) discretionary UPF consumption by demographic characteristics of parents (*n* = 2011).

	*n* (%)	UPF Consumption (serv/wk)	*p*-Value
		*mean (SD)*	
Age			<0.001
≤44 y	423 (21)	22.8 (12.5)	
≥45 y	1588 (79)	14.5 (8.4)	
Gender			0.804
Male	823 (41)	16.2 (9.4)	
Female	1188 (59)	16.3 (10.4)	
Education level			<0.001
Low	91 (5)	12.9 (8.6)	
Medium	750 (39)	13.3 (6.9)	
High	1093 (57)	18.6 (11.2)	
Family status			<0.001
Live alone	249 (12)	17.8 (11.3)	
Live with a partner	1716 (85)	15.9 (9.6)	
Live with others	46 (2)	22.2 (12.3)	
Family income			<0.001
<€2000	467 (27)	14.8 (8.5)	
€2000–4000	796 (47)	16.3 (9.6)	
>€4000	442 (26)	20.9 (11.9)	
Area of living			<0.001
Urban	1629 (81)	17.1 (10.4)	
Rural	382 (19)	12.8 (7.0)	

Abbreviations: Ultra-Processed Foods (UPFs).

**Table 3:** Children’s weekly mean (and standard deviation) UPF consumption by eating habits (*n* = 2011).

	*n* (%)	UPF Consumption (serv/wk)	*p*-Value
		*mean (SD)*	
Breakfast			<0.001
Never/seldom	277 (13.8)	19.5 (10.8)	
Often	348 (17.3)	23.4 (12.2)	
Always	1386 (68.9)	13.8 (7.9)	
Eating with family			<0.001
Seldom	41 (2.0)	18.1 (11.2)	
Often	601 (29.9)	22.6 (11.7)	
Daily	1369 (68.1)	13.4 (7.5)	
Eating alone			<0.001
Never/seldom	1247 (62.0)	12.9 (6.9)	
Often	597 (29.7)	22.2 (11.4)	
Daily	167 (8.3)	19.6 (12.4)	
Eating at school			<0.001
Never/seldom	831 (41.3)	13.7 (7.8)	
Often	622 (30.9)	20.2 (11.3)	
Almost daily	558 (27.7)	15.7 (9.9)	
Eating home-cooked food			<0.001
Seldom	252 (12.5)	12.2 (7.9)	
Often	891 (44.3)	20.3 (10.9)	
Almost daily	868 (43.2)	13.3 (7.7)	

Abbreviations: Ultra-Processed Food (UPF).

**Table 4:** Mean scores of parental food literacy (based on SFLQ score), perceived barriers and enablers (PBE score), healthy-eating attitudes (based on HEA score), and healthy-eating behaviour (based on TIUH score) by groups of discretionary UPF consumption (based on median weekly intake cut-off point).

	UPF Consumption		
	Low	High	*p*-Value
	*mean (SD)*		
SFLQ	31.5 (5.3)	31.9 (4.9)	0.07
PBE	65.5 (8.9)	61.5 (7.9)	<0.001
HEA	6.9 (1.8)	6.9 (1.9)	0.98
TIUH			
Health Info	2.8 (1.9)	3.8 (1.9)	<0.001
Consciousness	20.7 (3.1)	20.2 (3.2)	0.001
Orientation	30.9 (5.3)	31.5 (5.2)	0.02
Believes	31.8 (5.8)	30.9 (6.3)	0.001

Abbreviations: Healthy-eating Attitudes (HEA), Perceived Barriers and Enablers (PBE), Short Food Literacy Questionnaire (SFLQ), Theory of Internet Use related to Health (TIUH), Ultra-Processed Foods (UPF).

**Table 5:** Association between parental food literacy (based on SFLQ score), perceived barriers and enablers (PBE score), healthy-eating attitudes (based on HEA score), and healthy-eating behavior (based on TIUH score) and their children’s weekly consumption of discretionary UPF.

	UPF Consumption						
	SFLQ	PBE	HEA	TIUH			
				Health Info	Consciousness	Orientation	Beliefs
	*beta (SD)*						
Model 1	0.179 (0.044) **	−0.410 (0.042) **	0.240 (0.122) *	1.715 (0.104) **	−0.186 (0.069) *	0.186 (0.042) **	−0.099 (0.036) *
Model 2	0.247 (0.046) **	−0.356 (0.041) **	0.533 (0.124) **	1.577 (0.105) **	−0.047 (0.072)	0.280 (0.042) **	0.040 (0.047)
Model 3	0.248 (0.041) **	−0.228 (0.039) **	0.231 (0.112) *	1.063 (0.102) **	0.094 (0.064)	0.240 (0.037) **	0.127 (0.042) *
Model 4	0.138 (0.045) *	−0.217 (0.042) **	0.131 (0.118)	0.757 (0.115) **	−0.045 (0.068)	0.146 (0.041) **	0.022 (0.045)
Model 5	0.124 (0.046) *	−0.234 (0.042) **	0.111 (0.118)	0.743 (0.115) **	−0.069 (0.069)	0.136 (0.041) *	0.008 (0.046)

Abbreviations: Healthy-eating Attitudes (HEA), Perceived Barriers and Enablers (PBE), Short Food Literacy Questionnaire (SFLQ), Theory of Internet Use related to Health (TIUH), Ultra-Processed Foods (UPF). Model 1, unadjusted. Model 2, adjusted for children’s and adolescents’ age, gender, weight status, physical activity, sleep duration, and screen time. Model 3, adjusted for variables in model 2 + breakfast frequency, eating with family, eating alone, eating at school, and eating home-made foods. Model 4, adjusted for variables in model 3 + parents’ age, gender, education, income, family status, and area of living. Model 5, adjusted for variables in model 4 + adherence to the Mediterranean diet (based on KIDMED score). * *p* < 0.05, ** *p* < 0.001.

## Data Availability

The original contributions presented in the study are included in the article, further inquiries can be directed to the corresponding author.

## Abbreviations

The following abbreviations are used in this manuscript:

BMI	Body Mass Index
CDC	Centers for Disease Control and Prevention
HEA	Healthy-Eating Attitudes
HEAQ	Healthy-Eating Attitudes Questionnaire
IPAQ	International Physical Activity Questionnaire
PBE	Perceived Barriers and Enablers
SE	Standard Error
SFLQ	Short Food Literacy Questionnaire
SD	Standard Deviation
TIUH	Theory of Internet Use Related to Health
TPB	Theory of Planned Behavior
UPF	Ultra-Processed Foods
